# Comprehensive analysis of angiogenesis-related genes and pathways in early diabetic retinopathy

**DOI:** 10.1186/s12920-020-00799-6

**Published:** 2020-09-29

**Authors:** Chufeng Gu, Thashi Lhamo, Chen Zou, Chuandi Zhou, Tong Su, Deji Draga, Dawei Luo, Zhi Zheng, Lili Yin, Qinghua Qiu

**Affiliations:** 1 Department of Ophthalmology, Shanghai General Hospital, Shanghai Jiao Tong University School of Medicine, Shanghai, P.R. China; 2 National Clinical Research Center for Eye Diseases, Shanghai Key Laboratory of Ocular Fundus Diseases; Shanghai Engineering Center for Visual Science and Photomedicine; Shanghai engineering center for precise diagnosis and treatment of eye diseases, Shanghai, P.R. China; 3 Department of Ophthalmology, Shigatse People’s Hospital, Xizang, P.R. China; 4 Eye Institute, Eye and ENT Hospital, Shanghai Medical College, Fudan University, Shanghai, P.R. China

**Keywords:** Diabetic retinopathy, Angiogenesis, Bioinformatics analysis

## Abstract

**Background:**

Angiogenesis is an important parameter in the development of diabetic retinopathy (DR), and it is indicative of an early stage evolving into a late phase. Therefore, examining the role of angiogenic factors in early DR is crucial to understanding the mechanism of neovascularization.

**Methods:**

The present study identified hub genes and pathways associated with angiogenesis in early DR using bioinformatics analysis. Genes from published literature and Gene Expression Omnibus (GEO) were collected and analysed.

**Results:**

We collected 73 genes from 70 published studies in PubMed, which were referred to as DR-related gene set 1 (DRgset1). The gene expression profile of GSE12610 was downloaded, and 578 differentially expressed genes (DEGs) between diabetic and normal samples were identified. DEGs and DRgset1 were further combined to create DR-related gene set 2 (DRgset2). After an enrichment analysis, we identified 12 GO terms and 2 pathways associated with neovascularization in DRgset1, and 8 GO terms and 2 pathways in DRgset2. We found 39 new genes associated with angiogenesis and verified 8 candidate angiogenesis-related genes in DR cells using real-time PCR: *PIK3CB, ALDH3A1, ITGA7, FGF23, THBS1, COL1A1, MAPK13*, and *AIF1*. We identified 10 hub genes associated with neovascularization by constructing a protein-protein interaction (PPI) network: *TNF, VEGFA, PIK3CB, TGFB1, EDN1, MMP9, TLR4, PDGFB, MMP2,* and *THBS1.*

**Conclusions:**

The present study analysed angiogenesis-related genes and pathways in early DR in a comprehensive and systematic manner. *PIK3CB, ALDH3A1, ITGA7, FGF23, THBS1, COL1A1, MAPK13*, and *AIF1* may be the candidate genes to further explore the mechanisms of angiogenesis in early DR. *TNF, PIK3CB, TGFB1, EDN1, MMP9, TLR4, PDGFB, MMP2,* and *THBS1* may be new targets for early neovascularization therapy in the future.

## Background

Diabetic retinopathy (DR) is a highly specific microvascular complication of diabetes mellitus, and it is a leading cause of blindness in the working-age population globally [1]. Clinically, DR is divided into two stages, non-proliferative diabetic retinopathy (NPDR) and proliferative diabetic retinopathy (PDR), which representing an early and late stage, respectively [2, 3].

The emergence of new blood vessels is the chief symbolic event that is indicative of NPDR evolution into PDR [2]. Angiogenesis may further lead to vitreous haemorrhage, tractional retinal detachment, neovascular glaucoma, and blindness [4]. The mechanisms of neovascularization and vascular leakage are complicated, and vascular endothelial growth factor (VEGF) is the main angiogenic regulator [3, 5]. Angiogenic cytokines in the vitreous humour are altered in early DR. For example, transforming growth factor beta (TGF-β) and placental growth factor (PGF) are elevated in the aqueous humour of patients with NPDR [6]. The concentrations of angiopoietin 1 and 2 in patients with NPDR are higher than patients without DR, but the concentrations decrease in PDR patients [7]. Consequently, examination of the role of angiogenic factors in early DR plays an important role in understanding the mechanism of neovascularization.

Increasing evidence suggests that angiogenesis is associated with various biological processes and pathways in DR, such as the mitogen-activated protein kinase signaling pathway [8] and the tumour necrosis factor signaling pathway [9]. Animal experiments indicated that anti-angiogenic therapy in early DR may have a positive effect on the prognosis [10, 11]. However, these studies are isolated, and the role of angiogenesis in early DR is not fully understood. A comprehensive and systematic understanding of the molecular mechanisms of angiogenesis in NPDR would be conducive to therapeutic intervention.

**Fig. 1:**
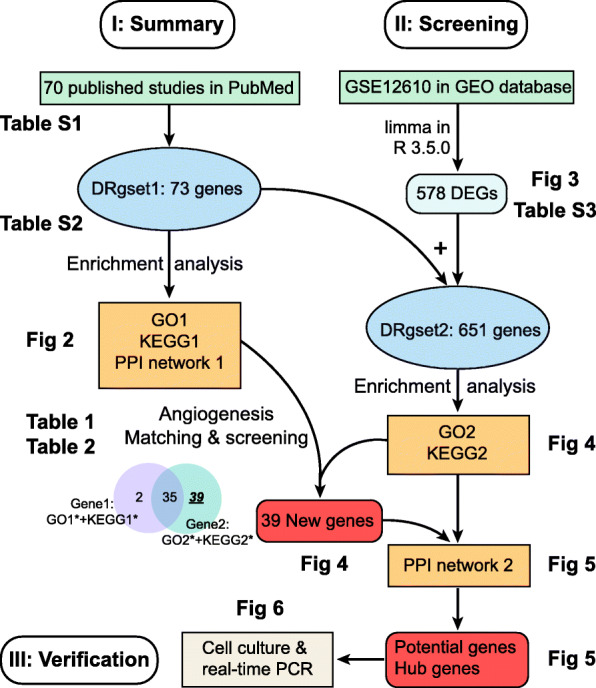
Flow chart for the comprehensive analysis of angiogenesis-related genes and pathways in early diabetic retinopathy. DRgset1, DR-related gene set 1; DRgset2, DR-related gene set 2; DEGs, differentially expressed genes; PPI, protein-protein interaction

The present study examined early DR-related biological processes, especially the process of neovascularization, by enriching the genes reported in the published literature and mined from a microarray in the Gene Expression Omnibus (GEO) database. Based on a summary of the published literature, we further explored new genes related to early DR using microarray data analysis. We found 39 new genes associated with angiogenesis in the early stage of DR from GEO using bioinformatics analysis and identified 8 candidate angiogenesis-related genes after verification. We also analysed the hub genes of neovascularization, which may serve as potential targets for the treatment of newly formed blood vessels. The flow chart for exploring the angiogenesis-related genes and pathways in early diabetic retinopathy is shown in Fig. 1.

## Methods

### Identification of genes from the literature

We identified early DR-related genes from the published literature in PubMed with the following search terms: (“Diabetic Retinopathy”) AND (“Non-Proliferative Diabetic Retinopathy” OR “NPDR” OR “early”) NOT (“clinical trial” OR “clinical trials as topic” OR “review” OR “review literature as topic”). Records were searched as of February 13, 2020 with no language restrictions.

To identify genes more comprehensively, a gene reported as significant to early DR in a study was included, even when no significance was indicated in other studies. Genes reported as associated with early DR from the published literature are referred to as DR-related gene set 1 (DRgset1). Gene names were uniformly converted into official gene symbols according to guidelines of the National Center of Biotechnology Information.

### Enrichment analysis

Gene ontology (GO) and the Kyoto Encyclopedia of Genes and Genomes (KEGG) pathway enrichment analyses of the DRgset1 were performed using the Database for Annotation, Visualization and Integrated Discovery (DAVID; version 6.8, https://david.ncifcrf.gov/) [12]. The GO terms and KEGG pathways with a false discovery rate (FDR) < 0.05 were considered significantly enriched and referred to as GO1 and KEGG1, respectively.

### PPI network construction

The Search Tool for the Retrieval of Interacting Genes/Proteins (STRING; version 11.0, https://string-db.org/) [13] was used to evaluate information on protein-protein interaction (PPI) in DRgset1. The network was constructed based on protein pairs with an interaction score of > 0.4, and Cytoscape 3.6.1 [14] was used to visualize this network in a more intuitive manner. Hub genes are identified using cytoHubba in Cytoscape 3.6.1 ranked by the Betweenness method.

### Identification of genes from GEO

#### Microarray data and quality assessment

The gene expression dataset GSE12610 from an early DR mouse study [15, 16] was downloaded from the GEO database (http://www.ncbi.nlm.nih.gov/geo/). GSE12610 contains 5 *Mus musculus* samples (CD1, adult, random sexes), including 3 diabetic samples (streptozotocin-induced diabetes for 5 weeks) and 2 normal samples. RNA was extracted from retinas. The platform used was the GPL1261 [Mouse430_2] Affymetrix Mouse Genome 430 2.0 Array.

We used relative log expression (RLE) and normalized unscaled standard errors (NUSE) to evaluate the quality of the microarray data using the R software (version 3.5.0, R Foundation for Statistical Computing, Vienna, Austria). If the quality is reliable in the RLE boxplot diagram, the centre of each sample would be close to the position of the ordinate 0, and the value of each sample in the NUSE diagram would be approximately 1.

#### Data preprocessing and identification of DEGs

The raw intensity values were background corrected, log2 transformed, and quantile normalized using the Robust Multi-array Average algorithm from the affy package in R 3.5.0. Subsequently, the probe IDs were converted to official gene symbols. If multiple probes corresponded to a single given gene, the average expression value of those probes was regarded as the value of this gene. The k-Nearest Neighbour was performed to fill in the missing value.

After the preprocessing, the differentially expressed genes (DEGs) between diabetic samples and normal samples were identified using the limma package in R 3.5.0. The Benjamini and Hochberg method was used for the adjustment of *P*-value, which is defined as FDR. The genes with fold change (FC) > 1.5 and FDR < 0.05 in GSE12610 were selected as DEGs. Volcano plot and heat maps were drawn in R 3.5.0 to visualize their expression values in different samples. DEGs and DRgset1 were combined to form DR-related gene set 2 (DRgset2).

### Identification of new genes and hub genes

Enrichment analyses of the DRgset1 in the published literature identified the GO terms and KEGG pathways related to the angiogenesis involved in early DR, which was confirmed in experiments. We performed enrichment analysis on the DRgset2 again to further examine new genes in these GO terms and pathways.

GO and KEGG pathway enrichment analyses of the DRgset2 were performed using DAVID. The GO terms and KEGG pathways with FDR < 0.05 were considered significantly enriched and referred to as GO2 and KEGG2, respectively. We obtained the intersection of GO1 and GO2 and KEGG1 and KEGG2 using Venn plots. The genes related to angiogenesis in the intersection of GO1 and KEGG1 were classified as Gene1, which was reported in the published literature. Similarly, we acquired Gene2, then excluded the published parts in Gene2 and obtained new genes, the unpublished portion of Gene2.

A PPI network was constructed for DRgset2 using STRING and Cytoscape. The top 100 nodes were selected using cytoHubba and ranked by the Betweenness method. The intersection of the Top100 and new genes were considered potential candidate genes related to angiogenesis in early DR. Similarly, the intersection of Top100 and Gene1 + Gene2 were considered potential angiogenesis-related hub genes in early DR.

### DR cell culture

Human retinal microvascular endothelial cells (HRMECs) were purchased from Applied Cell Biology Research Institute (Kirkland, WA, USA) and cultured in M199 medium supplemented with 20% foetal bovine serum [17]. Cells were exposed to normal glucose (5.5 mmol/L) and high glucose (30 mmol/L) for 48 h [16]. D-mannitol was used to form an equal level of osmotic pressure for all of the culture conditions.

### RNA extraction and real-time PCR

Total RNA was extracted from HRMECs using TRIzol reagent (Invitrogen, Carlsbad, CA, USA) and reverse transcribed into complementary DNA using PrimeScript™ RT Master Mix (TaKaRa, Japan) following the manufacturer’s protocol. Real-time PCR was performed using TB Green™ Premix Ex Taq™ (TaKaRa, Japan). The oligonucleotide primers that were used for the PCR amplifications were purchased from BioSune Biotechnology (Shanghai, China) and listed as follows:

*PIK3CB*: Forward, 5′-TATTTGGACTTTGCGACAAGACT-3′ and Reverse, 5′-TCGAACGTACTGGTCTGGATAG-3′;

*THBS1*: Forward, 5′-AGACTCCGCATCGCAAAGG-3′ and Reverse, 5′-TCACCACGTTGTTGTCAAGGG-3′;

*COL1A1*: Forward, 5′-GAGGGCCAAGACGAAGACATC-3′ and Reverse, 5′-CAGATCACGTCATCGCACAAC-3′;

*MAPK13*: Forward, 5′-TGAGAACGTCATTGGGCTCC-3′ and Reverse, 5′-AGCATCTGATACACCAGGTACT-3′;

*ALDH3A1*: Forward, 5′-TGGAACGCCTACTATGAGGAG-3′ and Reverse, 5′-GGGCTTGAGGACCACTGAG-3′;

*ITGA7*: Forward, 5′-CAGCGAGTGGACCAGATCC-3′ and Reverse, 5′-CCAAAGAGGAGGTAGTGGCTATC-3′;

*FGF23*: Forward, 5′-CAGAGCCTATCCCAATGCCTC-3′ and Reverse, 5′-GGCACTGTAGATGGTCTGATGG-3′;

*AIF1*: Forward: 5′-ATGAGCCAAACCAGGGATTTAC-3′ and Reverse, 5′-GGGATCGTCTAGGAATTGCTTGT-3′;

*DLL4*: Forward, 5′-GCCCTTCAATTTCACCTGGC-3′ and Reverse, 5′-CAATAACCAGTTCTGACCCACAG-3′;

*β-actin*: Forward, 5′-GCACCGCAAATGCTTCTA-3′ and Reverse, 5′-GGTCTTTACGGATGTCAACG-3′.

### Statistical analysis

The data in this study are presented as the means ± SD. Two-tailed Student’s t-test was used to compare the statistical significance between two groups. *P* < 0.05 was considered statistically significant.

## Results

### Identification of DRgset1 from the literature

We initially screened 3038 studies. The specific search results are given in the supplementary material (Additional file 1). Review of the abstracts led to the exclusion of 2608 studies as irrelevant to our topic. A total of 118 studies focused on DR or PDR, and 54 studies focused primarily on diabetic nephropathy. A total of 143 studies did not find the significant genes, and 45 studies were designed as “case reports.” Finally, we collected 73 DR-related genes (DRgset1) from 70 studies. A detailed table of all genes reported as associated with early DR is provided in supplementary material (Additional file 2).

### Enrichment analysis of DRgset1

**Fig. 2:**
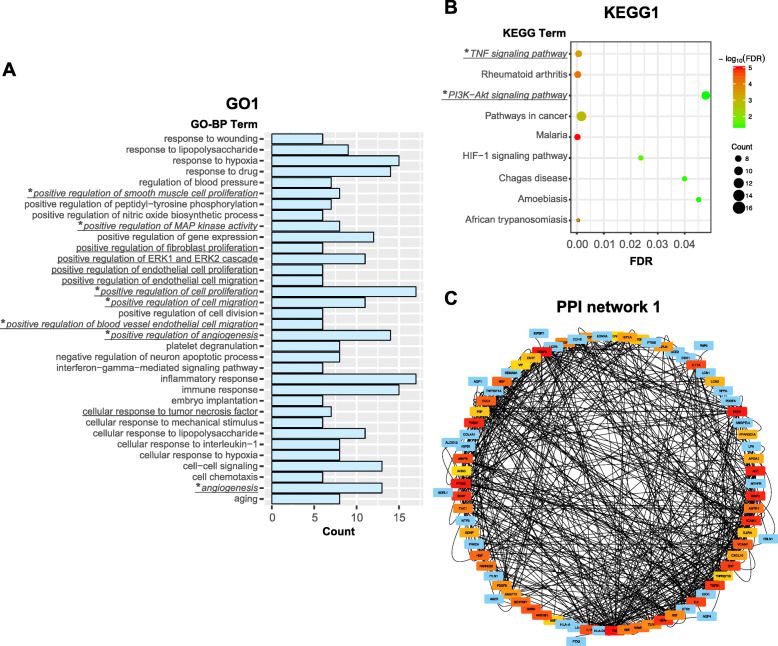
Enrichment analysis and PPI network of DRgset1. **a** GO1: the enriched GO-BP terms of DRgset1; **b** KEGG1: the KEGG pathways enriched in DRgset1; **c** The PPI network of DRgset1. DEGs, differentially expressed genes; PPI, protein-protein interaction; the underlined part represents the BP terms or pathways associated with angiogenesis; * represents the BP terms or pathways associated with angiogenesis shared by DRgset1 and DRgset2

Thirty-four enriched GO biological process (BP) terms (GO1, Fig. 2a) and 9 KEGG pathways (KEGG1, Fig. 2b) were identified. The biological processes and pathways of early DR are primarily related to hypoxia, inflammation, apoptosis, and angiogenesis. We found 12 terms associated with neovascularization among the 34 GO-BP terms (Fig. 2a) and 2 pathways among the 9 KEGG pathways (Fig. 2b). The PPI network (Fig. 2c) was constructed with 73 nodes and 603 edges. The top 50 nodes are marked using cytoHubba in Cytoscape 3.6.1 and ranked by Betweenness method.

### Identification of DEGs

**Fig. 3:**
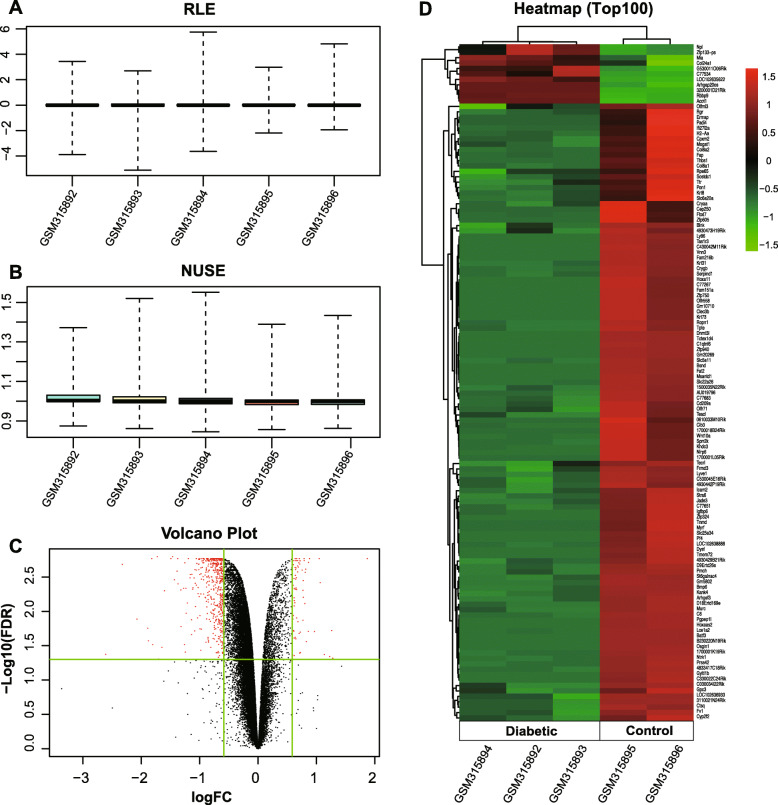
Identification of DEGs from GSE12610. **a** The RLE boxplot and **b** NUSE diagram for the quality of GSE12610; **c** the volcano plot of the expression of DEGs, red nodes represent DEGs; and **d** the heat map for the hierarchical cluster analysis of the top 100 DEGs, the red colour indicates high expression, and green indicates low expression. DEGs, differentially expressed genes; RLE, relative log expression; NUSE, normalized unscaled standard errors; and FC, fold change

The results of the quality assessment of GSE12610 were shown in Fig. 3a and b. In the RLE boxplot diagram (Fig. 3a), the centre of each sample is close to the position of the ordinate 0, and the value of each sample is approximately 1 in the NUSE diagram (Fig. 3b), which indicates that the quality of this dataset is reliable. There were 578 DEGs identified in diabetic samples compared to the normal samples, including 73 upregulated genes and 505 downregulated genes (DEGs, supplementary material, Additional file 3). The volcano plot and heat map, which revealed the distinct expression of DEGs, are presented in Fig. 3c and d, respectively.

### Enrichment analysis of DRgset2

**Fig. 4:**
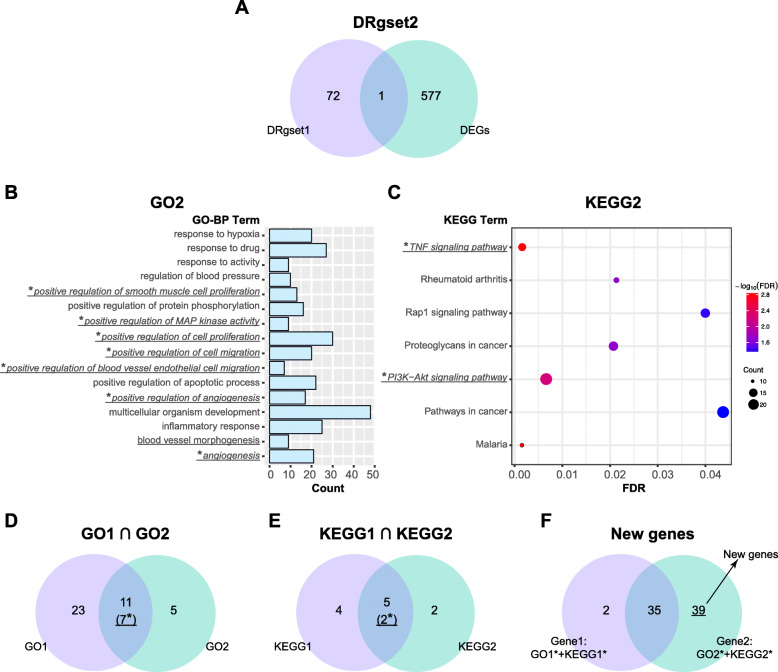
Enrichment analysis of DRgset2. **a** The Venn plot of DRgset1 and DEGs; **b** GO2: the enriched GO-BP terms of DRgset2; **c** KEGG2: the KEGG pathways enriched in DRgset2; **d** The Venn plot of GO1 and GO2; **e** The Venn plot of KEGG1 and KEGG2; and **f** The Venn plot of Gene1 and Gene2. Gene1 = GO1 + KEGG1; Gene2 = GO2 + KEGG2; New genes: the unpublished portion of Gene2; the underlined part represents the BP terms or pathways associated with angiogenesis; * represents the BP terms or pathways associated with angiogenesis shared by DRgset1 and DRgset2

DEGs and DRgset1 were combined to create DRgset2 (651 genes, Fig. 4a). We performed enrichment analysis on DRgset2 once again to further examine the new genes in the GO terms and pathways related to angiogenesis involved in early DR, which were identified in DRgset1 (Fig. 2).

Sixteen enriched GO-BP terms (GO2, Fig. 4b) and 7 KEGG pathways (KEGG2, Fig. 4c) were identified. The biological processes and pathways were also primarily related to hypoxia, inflammation, apoptosis, and angiogenesis. We found 8 terms associated with neovascularization among the 16 GO-BP terms (Fig. 4b) and 2 among the 7 KEGG pathways (Fig. 4c).

There were 7 terms associated with neovascularization in both GO1 and GO2 (Table 1, Fig. 4d, Fig. 2a* & Fig. 4b*), and 2 pathways in both KEGG1 and KEGG2 (Table 2, Fig. 4e, Fig. 2b* & Fig. 4c*). The genes related to angiogenesis at the intersection of GO1 and KEGG1 were classified as Gene1 (37 genes). Similarly, we obtained Gene2 (74 genes). Excluding the published part in Gene2, we acquired new genes (39 genes, Fig. 4f).

**Table 1:** GO-BP terms related to neovascularization shared by GO1 and GO2

GO-BP terms	Genes in GO1	Genes in GO2
positive regulation of smooth muscle cell proliferation	NAMPT, TNF, PDGFB, PTGS2, EDN1, PPARGC1A	PRKCA, NAMPT, TNF, PDGFB, PTGS2, AIF1, EDN1, AGER, PPARGC1A, HIF1A, VEGFA, THBS1, ALOX12
positive regulation of MAP kinase activity	EDN3, TNF, PDGFB, PDGFA, EDN1, VEGFA, TGFB1	EDN3, TNF, PDGFB, PDGFA, VEGFA, EDN1, TPD52L1, FGF1, TGFB1
positive regulation of cell proliferation	VIP, NAMPT, EDN3, NTF3, PDGFB, PGF, PDGFA, EDN1, HGF, GDNF, TGFB1, CXCL10, CNTF, VEGFA	EDN3, TNF, PTGS2, PDGFB, PGF, PDGFA, EDN2, EDN1, FGF10, GDNF, GHRHR, TGFB1, CXCL10, ALDH3A1, EDNRA, GADD45GIP1, THBS1, FGF1, TRAF5, APLN, NTF3, HGF, PLAC8, OSM, PRAMEF12, CNTF, HIF1A, VEGFA, ALOX12, NGF
positive regulation of cell migration	PRKCA, NTF3, PDGFB, PDGFA, SEMA3E, EDN1, VEGFA, HGF, TGFB1	PRKCA, PDGFB, NTF3, PDGFA, AIF1, EDN1, HGF, AQP1, MMP2, AGER, TGFB1, CXCL10, VEGFA, SEMA3E, SEMA3C, COL1A1, THBS1, FGF1, MYOC, ALOX12
positive regulation of blood vessel endothelial cell migration	PRKCA, PDGFB, VEGFA, HSPB1, ANGPT1, TGFB1	PRKCA, PDGFB, VEGFA, HSPB1, ANGPT1, THBS1, TGFB1
positive regulation of angiogenesis	PRKCA, UTS2, PGF, CXCL8, HGF, AQP1, PTGIS, HIF1A, VEGFA, HSPB1, ANGPT2, ANGPTL4	PRKCA, UTS2, PGF, MMP9, C6, HGF, AQP1, TNFRSF1A, HIF1A, PTGIS, VEGFA, HSPB1, UTS2R, FGF1, THBS1, ANGPT2, ALOX12
angiogenesis	PRKCA, HIF1A, CCL2, PTGS2, PDGFA, PGF, VEGFA, CXCL8, ANGPT1, ANGPT2, ANGPTL4	PRKCA, COL4A1, CCL2, PTGS2, PDGFA, PGF, FGF10, MMP2, HOXB3, TAL1, HIF1A, DLL4, FAP, VEGFA, SEMA3E, ANGPT1, COL8A1, FGF1, COL8A2, ANGPT2, ANGPTL4

**Table 2:** KEGG pathways related to neovascularization shared by KEGG1 and KEGG2

KEGG pathway	Genes in KEGG1	Genes in KEGG2
TNF signaling pathway	VCAM1, ICAM1, TNFRSF1A, TNFRSF1B, TNF, CCL2, PTGS2, MMP9, EDN1, CXCL10	ICAM1, TNF, CCL2, PTGS2, PIK3CB, MMP9, EDN1, IFI47, CXCL10, VCAM1, TNFRSF1A, TNFRSF1B, MAPK13, TRAF5
PI3K-Akt signaling pathway	PRKCA, IL2RA, PDGFB, PDGFA, PGF, VEGFA, TLR4, ANGPT1, HGF, ANGPT2, NGF	PRKCA, COL4A1, IL2RA, PDGFB, PDGFA, PGF, PIK3CB, TCL1B4, FGF23, FGF10, TLR4, HGF, OSM, CCNE2, VEGFA, ITGA7, ANGPT1, COL1A1, THBS1, FGF1, COL24A1, ANGPT2, THBS4, NGF

### Identification of new genes and hub genes

**Fig. 5:**
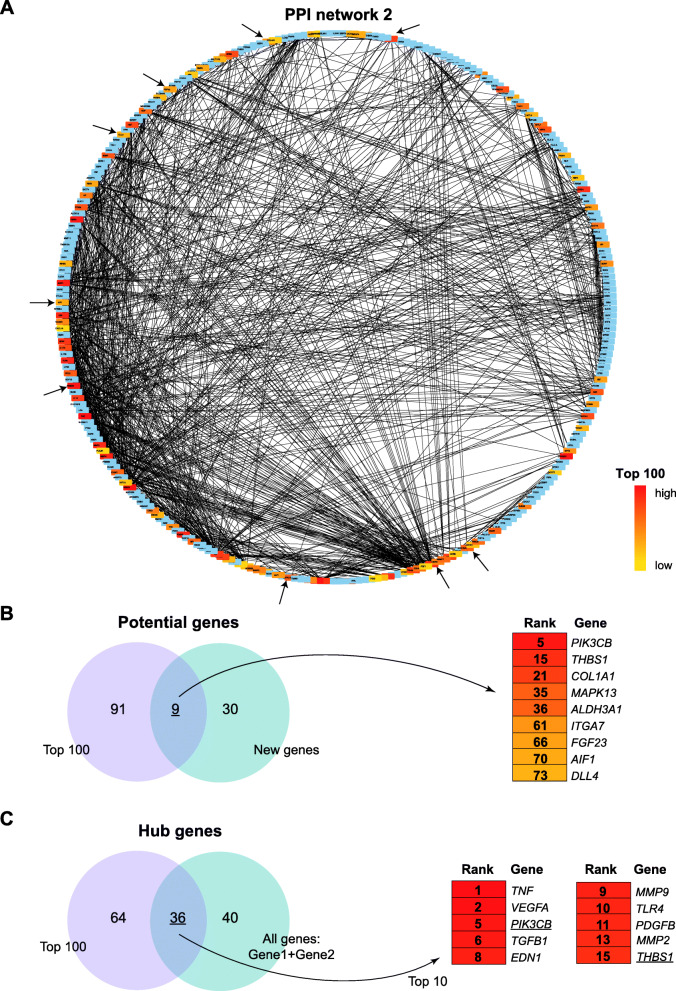
Identification of new genes and hub genes. **a** The PPI network of DRgset2. The top 100 genes are marked using cytoHubba; **b** New potential genes related to angiogenesis in early DR; **c** Angiogenesis-related hub genes in early DR. Gene1 = GO1 + KEGG1; Gene2 = GO2 + KEGG2; New genes: the unpublished portion of Gene2

The PPI network of DRgset2 (Fig. 5a) was constructed with 269 nodes and 1211 edges. The top 100 genes were marked using cytoHubba and ranked by the Betweenness method. After considering the intersection of the top 100 genes and new genes, we found 9 potential candidate genes related to angiogenesis in early DR (Fig. 5b): *PIK3CB, THBS1, COL1A1, MAPK13, ALDH3A1, ITGA7, FGF23, AIF1,* and *DLL4*. At the intersection of the top 100 genes and Gene1 + Gene2, we identified the top 10 genes, which may be potential hub genes related to angiogenesis in early DR (Fig. 5c): *TNF, VEGFA, PIK3CB, TGFB1, EDN1, MMP9, TLR4, PDGFB, MMP2,* and *THBS1.*

### Verification of candidate genes

**Fig. 6:**
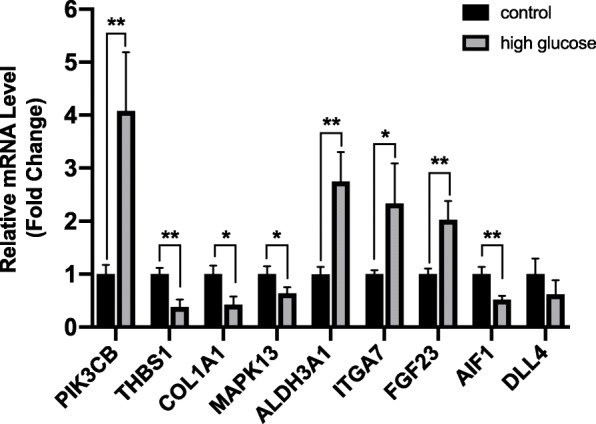
Real-time PCR analysis of candidate genes in early DR (HRMECs). * *p* < 0.05; ** *p* < 0.01

Real-time PCR was performed to detect the expression of mRNA of candidate genes. As shown in Fig. 6, four genes were significantly overexpressed, *PIK3CB, ALDH3A1, ITGA7*, and *FGF23*, and four genes were significantly under-expressed, *THBS1, COL1A1, MAPK13*, and *AIF1*. The results of PCR were highly consistent with the microarray results (supplementary material, Additional file 3). We will verify the functions of these 8 novel angiogenesis-related genes in our future studies.

## Discussion

DR is one of the most complex, heterogeneous, and multifactorial clinical disorders. Angiogenesis is one of the critical events in the progression of DR. Hyperglycaemia may cause an imbalance between stimulators and inhibitors of angiogenesis, which further leads to the formation of new blood vessels [18]. These immature blood vessels are more prone to leakage and ultimately result in serious complications [19]. Antiangiogenic treatment provides significant improvements in disease conditions. Intravitreal injection of anti-VEGF drugs is a major treatment for PDR [3, 20]. If one could manage these angiogenesis-related factors before the development of new blood vessels in the early stage, it would delay the progress of NPDR to PDR. Consequently, a full understanding of the angiogenic regulators in DR prior to the formation of new blood vessels is particularly significant. The present study performed a systematic examination of angiogenic regulators in early DR using bioinformatics analysis.

We identified the GO terms and KEGG pathways related to angiogenesis involved in early DR via enrichment analysis of the DRgset1 in the published literature, which were confirmed in experiments. We performed enrichment analysis on DRgset2 again to further examine the new genes in these GO terms and pathways. The enrichment analysis found 12 terms associated with neovascularization among the GO1 and 2 among KEGG1 (Fig. 2a and b), and 8 GO BP terms and 2 pathways in GO2 and KEGG2, respectively (Fig. 4b and c). This demonstrates the importance of neovascularization in the early stage of DR. There were 7 common BP terms (Fig. 4d) and 2 common pathways (Fig. 4e) related to angiogenesis both in the DRgset1 and DRgset2. We found 39 new genes associated with angiogenesis after excluding the published part of DRgset2. We detected 9 new potential genes related to angiogenesis among the top 100 genes in the PPI network of DRgset2 (Fig. 5b) and discovered 8 candidate angiogenesis-related genes in HRMECs using real-time PCR (Fig. 6): *PIK3CB, ALDH3A1, ITGA7, FGF23, THBS1, COL1A1, MAPK13*, and *AIF1*.

*PIK3CB*, phosphatidylinositol-4,5-bisphosphate 3-kinase catalytic subunit beta, is a major regulator of the PI3K/Akt pathway [21], and it takes part in the growth and metastasis of a variety of tumours [21, 22]. *PIK3CB* is involved in brain insulin resistance [23]. *ALDH3A1*, aldehyde dehydrogenase 3 family member A1, plays an important role in the metabolism of aldehydes and cellular oxidative stress-related processes [24]. *ITGA7*, integrin subunit alpha 7, participates in cell migration, morphological development, and metastasis [25]. *FGF23*, fibroblast growth factor 23, possesses broad mitogenic and cell survival activities, and it is implicated in hypophosphatemia [26] and diabetic nephropathy [27]. *THBS1*, thrombospondin 1, encodes an adhesive glycoprotein that is involved in angiogenesis and tumourigenesis [28]. *COL1A1*, collagen type I alpha 1 chain, is related to diabetic kidney disease [29]. *MAPK13*, mitogen-activated protein kinase 13, is involved in various processes, such as inflammation and apoptosis [30]. *AIF1*, allograft inflammatory factor 1, encodes a cytoskeleton-associated protein, and it is involved in the proliferation and migration of macrophages [31]. However, the functions of these genes in DR were not reported to date. The results of our study suggest that *PIK3CB*, *ITGA7*, *FGF23*, and *THBS1* mediate neovascularization in early DR via the PI3K-Akt signaling pathway. *COL1A1* and *AIF1* may mediate neovascularization via the regulation of the cell migration.

Angiogenesis is an the adverse factors in the progression of DR, and targeting it would help reduce the visual impairment caused by DR. Anti-VEGF treatment is the main therapy for the inhibition of neovascularization [32]. We identified 10 hub genes associated with neovascularization: *TNF, VEGFA, PIK3CB, TGFB1, EDN1, MMP9, TLR4, PDGFB, MMP2,* and *THBS1*. *TNF*, tumour necrosis factor, activates angiogenic factors, such as endothelin (*EDN*) and matrix metalloproteinases (*MMPs*), to mediate the progression of angiogenesis [33]. The overexpression of *EDN1* may lead to the aberrant haemodynamics in the early DR. [34] The results of our study suggest that *TNF* and *EDN1* jointly participate in the processes of cell proliferation, regulation of MAP kinase activity, and TNF signaling pathway. *MMP9* and *MMP2*, which were involved in the process of cell migration and TNF signaling pathway in our study, were observed increased in the retina and vitreous in DR and may increase the vascular permeability through disruption of tight junction complexes [35]. One report revealed that *TLR4*, toll-like receptor 4, also regulated tight junctions via TLR4/PI3K/Akt/JNK1/2/14–3-3ε/NF-κB/MMP9 pathway [36]. Therefore, we suspect that the *MMP9-*, *MMP2-* and *TLR4-* mediated signaling pathways may play an important roles in the cell tight junctions of DR. *TGFB1*, transforming growth factor beta 1, promotes the recruitment of endothelial cells and proliferation [37], which are elevated in DR. [6] *PDGFB*, platelet-derived growth factor subunit B, also regulates the proliferation and migration of pericytes via the TGFB pathway [38]. The results of our study suggest that *PDGFB* and *TGFB1* regulate cell proliferation and migration via the PI3K-Akt signaling pathway. Therefore, our data indicate that these genes may be new targets for early neovascularization therapy in the future.

To the best of our knowledge, this research is the first full-scale study to combine genes from the published literature and DEGs from data mining and examines the role of angiogenesis in early DR using bioinformatics technologies. However, there are several limitations in this study. First, genes that were once reported as significant in a previous study were included here for a comprehensive analysis, which may increase false-positive findings. Second, the sample size of the dataset GSE12610 is small. We found some datasets that included large sample size during data screening, but we did not include these data due to the poor data quality. Third, because the genes in mice and humans are highly conserved, we combined the DEGs found in the microarray and genes collected from the published literature. However, some of these genes may be heterogeneous. The filtering process found some human samples data, but we did not include these data because they used fibrovascular membranes of PDR. Fourth, the culture of a single type of cells is different from the in vivo environment to some extent. The construction of multiple types of cells in co-cultures and studies in vivo are required to confirm our findings.

## Conclusion

Angiogenesis is an important parameter in the early stage of DR. *PIK3CB, ALDH3A1, ITGA7, FGF23, THBS1, COL1A1, MAPK13*, and *AIF1* may be candidate genes for further examination in the mechanisms of angiogenesis in early DR. *TNF, PIK3CB, TGFB1, EDN1, MMP9, TLR4, PDGFB, MMP2,* and *THBS1* may be new targets to consider for early neovascularization therapy in the future. Bioinformatics methods help our understanding of the different biological processes in a comprehensive and systematic manner. However, examination of the molecular mechanisms of angiogenesis in early DR remains an ongoing task, and further animal and clinical studies are required to confirm our findings.

## Supplementary information

**Additional file 1: Table S1.** Specific search results of early DR-related literature.

**Additional file 2: Table S2.** Genes associated with early DR in literature (DRgset1).

**Additional file 3: Table S3.** DEGs associated with early DR from GSE12610.

## Data Availability

All data generated or analysed during this study are included in this published article and its supplementary information files. The raw matrix datasets can be downloaded from the Gene Expression Omnibus (GEO) database by searching “GSE12610” (https://www.ncbi.nlm.nih.gov/geo/query/acc.cgi?acc=GSE12610). GPL1261 annotation files can be required from the website: https://www.ncbi.nlm.nih.gov/geo/query/acc.cgi?acc=GPL1261.

## Abbreviations

AIF1: Allograft inflammatory factor 1
ALDH3A1: Aldehyde dehydrogenase 3 family member A1
BP: Biological process
COL1A1: Collagen type I alpha 1 chain
DAVID: Database for Annotation, Visualization and Integrated Discovery
DEGs: Differentially expressed genes
DR: Diabetic retinopathy
DRgset1: DR-related gene set 1
DRgset2: DR-related gene set 2
EDN: Endothelin
FC: Fold change
FDR: False discovery rate
FGF23: Fibroblast growth factor 23
GEO: Gene Expression Omnibus
GO: Gene ontology
HRMECs: Human retinal microvascular endothelial cells
ITGA7: Integrin subunit alpha 7
KEGG: Kyoto Encyclopedia of Genes and Genomes
MAPK13: Mitogen-activated protein kinase 13
MMPs: Matrix metalloproteinases
NPDR: Non-proliferative diabetic retinopathy
NUSE: Normalized unscaled standard errors
PDGFB: Platelet-derived growth factor subunit B
PDR: Proliferative diabetic retinopathy
PGF: Placental growth factor
PIK3CB: Phosphatidylinositol-4,5-bisphosphate 3-kinase catalytic subunit beta
PPI: Protein-protein interaction
RLE: Relative log expression
STRING: Search Tool for the Retrieval of Interacting Genes/Proteins
TGFB1: Transforming growth factor beta 1
TGF-β: Transforming growth factor beta
THBS1: Thrombospondin 1
TLR4: Toll-like receptor 4
TNF: Tumor necrosis factor
VEGF: Vascular endothelial growth factor
