# Supplementary material for: Comprehensive analysis of angiogenesis-related genes and pathways in early diabetic retinopathy
Source: BMC Med Genomics. 2020 Sep 29;13:142. doi: 10.1186/s12920-020-00799-6 (PMC7526206; doi:10.1186/s12920-020-00799-6)
Supplement: Supplementary file 1 — Additional file 1: Table S1. Specific search results of early DR-related literature. [file 12920_2020_799_MOESM1_ESM.pdf]

Table S1. Specific search results of early DR-related literature

Search	Query	Items found
#1	Diabetic Retinopathy[Mesh]	24041
#2	Non Proliferative Diabetic Retinopathy[Title/Abstract]) OR NPDR[Title/Abstract] OR early[Title/Abstract]	1478058
#3	"clinical trial"[Publication Type] OR "clinical trials as topic"[MeSH]	1108109
#4	"review"[Publication Type] OR "review literature as topic"[MeSH]	2624983
#5	#1 and #2 not #3 not #4	3038

Records were searched as of February 13, 2020, with no language restrictions.
