# Supplementary material for: Comprehensive analysis of angiogenesis-related genes and pathways in early diabetic retinopathy
Source: BMC Med Genomics. 2020 Sep 29;13:142. doi: 10.1186/s12920-020-00799-6 (PMC7526206; doi:10.1186/s12920-020-00799-6)
Supplement: Supplementary file 2 — Additional file 2: Table S2. Genes associated with early DR in literature (DRgset1). [file 12920_2020_799_MOESM2_ESM.pdf]

Table S2. Genes associated with early DR in literature (DRgset1)

Gene symbol	Gene name	Reference*
Ace	angiotensin-converting enzyme	26658948
Ager	advanced glycosylation end-product specific receptor	20685137；16043866
Agtr1	angiotensin II receptor type 1	10576062
Ahsg	alpha 2-HS glycoprotein	26110737
Angpt1	Angiopoietin 1	15774928
Angpt2	Angiopoietin 2	15774928
Angptl4	angiopoietin-like 4	25687026
Aqp1	aquaporin 1	26087356
Aqp4	aquaporin 4	26087356
B2m	beta-2-microglobulin	22040812
Bdnf	brain-derived neurotrophic factor	26004392
Ccl2	C-C motif chemokine ligand 2	28336215；25981750；23010641；21121809
Cntf	ciliary neurotrophic factor	29084332
Cxcl10	C-X-C motif chemokine ligand 10	21121809
Cxcl8	C-X-C motif chemokine ligand 8	29084332；21121809；11988817
Edn1	endothelin 1	28548649；20664700；18167288；16080914；15331199
Edn3	endothelin 3	15331199
Ednra	endothelin receptor type A	10907123
Fbln1	fibulin-1	27678201
Gdnf	glial cell-derived neurotrophic factor	29084332；22266674
Gfap	glial fibrillar acidic protein	23335850；
Gsr	glutathione-disulfide reductase	22653543
Hgf	hepatocyte growth factor	16324700
Hif1a	hypoxia inducible factor 1 subunit alpha	17162888
Hla-a	major histocompatibility complex, class I, A	9201596
Hla-b	major histocompatibility complex, class I, B	9201596
Hla-drb1	major histocompatibility complex, class II, DR beta 1	9201596
Hspb1	heat shock protein family B member 1	22040812
Icam1	intercellular adhesion molecule 1	14574977；25324196
Ido1	indoleamine 2, 3-dioxygenase 1	22034910
Il10	interleukin 10	29382585
Il17a	interleukin 17A	26940815
Il2ra	interleukin 2 receptor subunit alpha	11988817
Itln1	intelectin 1	26420914
Lcn1	lipocalin 1	22040812
Lcn2	lipocalin 2	27100138
Lpa	lipoprotein (a)	18700887
Mmp9	matrix Metalloproteinase 9	28964682

Mthfr	methylenetetrahydrofolate reductase	25324196；18774994
Naglu	N-acetyl-alpha-glucosaminidase	16696964
Nampt	nicotinamide phosphoribosyltransferase	25524991
Ngf	nerve growth factor	29084332
Nppa	natriuretic peptide A	15789000
Ntf3	neurotrophins-3	29084332
Nucb2	nucleobindin 2	28079928
Pdgfa	platelet-derived growth factor subunit A	19799585
Pdgfb	platelet-derived growth factor subunit B	19799585
Pedf	pigment epithelium-derived factor	12208245
Pgf	placenta growth factor	27678201
Ppargc1a	peroxisome proliferative activated receptor, gamma, coactivator 1 alpha	25324196
Prkca	protein kinase C alpha	15259294
Ptges	prostaglandin E synthase	10223650
Ptgis	prostaglandin I2 synthase	10223650
Ptgs2	prostaglandin-endoperoxide synthase 2	29408756
Ptx3	pentraxin-3	28574494
Rarres2	retinoic acid receptor responder 2	25848840
Rbp3	retinol binding protein 3	19823802
Sema3a	semaphorin 3A	27273597
Sema3e	semaphorin 3E	25687026
Sord	sorbitol dehydrogenase	18289528
Sorl1	sortilin related receptor 1	23652469
Sst	somatostatin	17704349
Tac1	tachykinin precursor 1	11274084
Tgfb1	transforming growth factor-beta1	17162922；27678201；16324700
Tlr4	toll-like receptor 4	19135114
Tnf	tumor necrosis factor	19135114；29084332；17641733；11988817；16696964
Tnfrsf1a	tumor necrosis factor receptors 1	22330960
Tnfrsf1b	tumor necrosis factor receptors 2	22330960
Tsp1	thrombospondin 1	17117553
Uts2	urotensin-II	22587369
Vcam1	vascular cellular adhesion molecule-1	14574977
Vegfa	vascular endothelial growth factor A	27286364；25687026；19799585；20424229；20444917；19263526；17849138；15905870；15259294；15331199；11527957；16324700；24502825
Vip	vasoactive intestinal polypeptide	11274084

* References are designated by PMIDs, which are the identifiers of PubMed. PMIDs in each gene term are equal and there’re not priorities in the permutation of the corresponding PMIDs of specified genes.
