# Supplementary material for: Comprehensive analysis of angiogenesis-related genes and pathways in early diabetic retinopathy
Source: BMC Med Genomics. 2020 Sep 29;13:142. doi: 10.1186/s12920-020-00799-6 (PMC7526206; doi:10.1186/s12920-020-00799-6)
Supplement: Supplementary file 3 — Additional file 3: Table S3. DEGs associated with early DR from GSE12610. [file 12920_2020_799_MOESM3_ESM.pdf]

Table S3. DEGs associated with early DR from GSE12610

Gene	LogFC	*P*-Value	Gene	LogFC	*P*-Value	Gene	logFC	*P*-Value
Dnmt3l	-1.72	9.86E-08	Crygb	-1.08	4.26E-05	4833444G19Rik	-0.61	6.61E-04
Tctex1d4	-1.46	1.54E-07	Osm	-0.69	4.28E-05	Prom2	-0.61	6.61E-04
Pgpep1l	-1.37	3.06E-07	AU019796	-1.01	4.30E-05	Blnk	-0.85	6.69E-04
3200001D21Rik	1.05	4.62E-07	BC025933	-0.70	4.30E-05	Rsph6a	-0.59	6.77E-04
C6	-1.19	5.01E-07	AW061096	-0.83	4.44E-05	Banf2	-0.75	6.82E-04
C1qtnf6	-1.49	5.47E-07	Shc3	-0.59	4.52E-05	Fam78a	-0.64	6.95E-04
Rbbp9	1.87	5.92E-07	Pctp	-0.76	4.55E-05	AW557046	0.63	7.00E-04
B230220N19Rik	-0.93	7.70E-07	Tmem252	-0.84	4.57E-05	Cyp2f2	-1.21	7.16E-04
Lce1a2	-0.97	8.48E-07	1500035N22Rik	-1.06	4.66E-05	Fbxw19	0.80	7.40E-04
Hoxaas2	-0.93	9.38E-07	Acsbg2	-0.69	4.74E-05	5330426L24Rik	-0.61	7.43E-04
Batf3	-0.86	1.04E-06	Ptprcap	-0.77	4.76E-05	4930448E22Rik	0.59	7.43E-04
Zfp940	-1.01	1.05E-06	4930444M15Rik	-0.66	4.82E-05	3110079O15Rik	-0.66	7.50E-04
Gm5802	-1.82	1.07E-06	Cenpp	-0.67	4.82E-05	Gnb1l	-0.76	7.65E-04
Osgin1	-0.85	1.16E-06	AU022255	0.76	4.92E-05	Fut1	-0.80	7.65E-04
Gm20269	-0.87	1.44E-06	Pm20d1	-0.60	4.92E-05	Tcl1b4	-0.60	7.67E-04
5830448L01Rik	0.79	1.49E-06	Matn4	-0.64	5.00E-05	E330013P04Rik	-0.64	7.78E-04
Bsnd	-0.94	1.83E-06	Tal1	-0.66	5.13E-05	Gm11545	-0.60	7.87E-04
Acot1	0.88	1.84E-06	Tmem210	-0.64	5.15E-05	Dbt	-0.64	8.00E-04
Lhfpl5	-0.74	1.94E-06	Gkn3	-0.62	5.17E-05	Dnah17	-0.69	8.05E-04
Fat2	-1.06	2.05E-06	Egr4	-0.76	5.29E-05	Slc7a10	-0.64	8.07E-04
Dusp23	-0.79	2.05E-06	Gm4640	-0.84	5.32E-05	AY761184	-0.73	8.31E-04
1700001K19Rik	-0.88	2.08E-06	Kcnu1	0.59	5.36E-05	Usp27x	-0.71	8.36E-04
Rtdr1	-0.73	2.09E-06	Dysf	-0.89	5.36E-05	Alg13	0.62	8.59E-04
Mapk13	-0.81	2.34E-06	S100a3	-0.70	5.37E-05	C79743	-0.70	8.60E-04
C80893	0.71	2.42E-06	Saysd1	-0.64	5.39E-05	Trim30a	-0.62	8.60E-04
Nnmt	-0.78	2.48E-06	Gm11738	-0.73	5.49E-05	Avil	-0.65	8.65E-04
Prss50	-0.76	2.86E-06	Klk11	0.59	5.54E-05	Olfr71	-1.07	8.93E-04
AB124611	-0.69	3.05E-06	Oxct2a	-0.62	5.68E-05	Thbs1	-0.88	9.31E-04
Bmp10	-0.70	3.07E-06	3110021N24Rik	-1.39	5.73E-05	Reg4	-0.68	9.34E-04
Tceal7	-0.67	3.12E-06	Zbtb38	-0.59	5.80E-05	LOC664787	-0.65	9.47E-04
D4Ertd669e	-0.73	3.15E-06	D630036G22Rik	-0.60	5.88E-05	4930428O21Rik	-0.62	9.56E-04
4930415O20Rik	-0.73	3.25E-06	Tpte	-0.86	6.11E-05	Acot3	-0.64	1.01E-03
Ntrk1	-0.94	3.34E-06	Gm16491	-0.68	6.12E-05	C79123	-0.74	1.05E-03
Gfod1	-0.72	3.37E-06	Fmo4	0.68	6.14E-05	Dok1	-0.62	1.06E-03
Il12rb1	-0.71	3.47E-06	Fbxo46	-0.71	6.21E-05	Adcy4	-0.64	1.09E-03
Ccdc185	-0.65	3.50E-06	Tmem72	-0.89	6.37E-05	Mmel1	-0.77	1.11E-03
Cidea	-0.65	3.55E-06	Fgf23	0.81	6.40E-05	4930449E01Rik	-0.63	1.12E-03
Chst13	0.64	3.81E-06	Serpind1	-0.89	6.41E-05	4930452G13Rik	-0.75	1.13E-03
4930468A15Rik	-0.69	3.93E-06	Chrne	-0.74	6.48E-05	Pcsk9	-0.62	1.17E-03

Zdhhc25	-0.71	3.95E-06	Hpn	-0.63	6.58E-05	LOC100049077	-0.77	1.18E-03
4930554P06Rik	-0.63	3.96E-06	Actrt2	-0.69	6.81E-05	Zfp575	-0.59	1.22E-03
AW125646	-0.68	4.03E-06	B930059L03Rik	-0.63	6.95E-05	1700016A09Rik	-0.67	1.25E-03
D8Ertd124e	-0.63	4.10E-06	Olfr558	-0.98	7.26E-05	4833428L15Rik	-0.61	1.30E-03
Pramef12	-0.62	4.14E-06	Bmp8a	-0.66	7.43E-05	Inhbc	-0.81	1.30E-03
Slc34a3	-0.63	4.16E-06	Itgam	-0.61	7.52E-05	Nhlh1	-0.68	1.30E-03
AI447881	-0.72	4.18E-06	Cyp2c68	-0.69	7.59E-05	Ankrd2	-0.60	1.32E-03
Hyal3	-0.62	4.22E-06	Clec3b	-1.13	7.65E-05	Gpc3	-1.23	1.40E-03
Nsun7	-0.66	4.26E-06	LOC102638888	-1.16	7.95E-05	D630030B22Rik	-0.61	1.41E-03
Hcrtr2	-0.66	4.37E-06	Krt73	-1.00	7.96E-05	B230303O12Rik	-0.79	1.41E-03
2310001K24Rik	-0.62	4.40E-06	D430018E03Rik	-0.69	8.00E-05	0610033M10Rik	-0.87	1.42E-03
Acr	-0.62	4.43E-06	Gm10710	-0.91	8.65E-05	Gm16740	-0.76	1.46E-03
Gm10421	-0.72	4.45E-06	Cyp2c38	0.64	8.91E-05	Agpat9	-0.64	1.46E-03
Txndc8	-0.65	4.53E-06	Slc25a34	-0.85	9.19E-05	Nrg2	-0.66	1.49E-03
Ntn5	-0.66	4.61E-06	Mfap4	-0.66	9.23E-05	Slc10a6	-0.59	1.49E-03
4930414N06Rik	-0.75	4.65E-06	C77798	-0.60	9.25E-05	Grik4	-0.75	1.50E-03
Ccdc68	0.66	4.68E-06	Birc7	-0.63	9.61E-05	Plaur	-0.60	1.59E-03
Txndc2	-0.79	4.70E-06	Gm16793	-0.85	9.64E-05	Lct	0.60	1.62E-03
Prss28	-0.66	4.88E-06	Exoc3l4	-0.63	9.71E-05	Col8a1	-0.99	1.63E-03
Endou	-0.60	4.96E-06	Cdca7	-0.59	9.77E-05	2610028H24Rik	-0.59	1.68E-03
D8Ertd51e	-0.82	5.15E-06	Aif1	-0.62	9.87E-05	Perp	-0.70	1.68E-03
Lrrn2	-0.62	5.17E-06	5033423O07Rik	-0.80	1.00E-04	Gm10804	-0.76	1.68E-03
Prss42	-0.92	5.30E-06	C77683	-0.94	1.00E-04	Spint4	-0.63	1.71E-03
2010003K11Rik	-0.79	5.86E-06	Fam151a	-0.88	1.01E-04	Tdrd1	-0.73	1.79E-03
Slc5a11	-0.87	5.88E-06	Fam115c	-0.70	1.02E-04	Sostdc1	-1.04	1.89E-03
B230205o20rik	-0.63	5.92E-06	E530011L22Rik	-0.72	1.04E-04	Itga7	0.66	1.89E-03
4931402H11Rik	-0.63	5.92E-06	Cdk1	-0.79	1.05E-04	5430440P10Rik	-0.59	1.91E-03
4833417C18Rik	-1.01	6.23E-06	Zfp750	-0.87	1.06E-04	Il17b	-0.77	1.91E-03
Pidd1	-0.64	6.31E-06	Prp2	0.66	1.10E-04	Rbm20	-0.59	2.00E-03
Srp54b	-0.74	6.32E-06	D7Ertd661e	-0.84	1.11E-04	4930473H19Rik	-0.88	2.01E-03
Msantd1	-1.00	6.33E-06	4930429B21Rik	-1.16	1.12E-04	Il1r2	0.60	2.02E-03
Cd300a	-0.71	6.50E-06	Ctla4	-0.66	1.12E-04	Col8a2	-2.07	2.03E-03
Hmcn1	-0.62	6.56E-06	Ctsq	-1.09	1.13E-04	Ghrhr	-0.59	2.04E-03
Esr2	-0.62	6.71E-06	Chst5	-0.60	1.15E-04	Tfap4	-0.68	2.05E-03
2410012E07Rik	-0.77	6.78E-06	Traf5	-0.61	1.19E-04	Tmem156	-0.71	2.06E-03
4731417B20Rik	-0.81	6.81E-06	Zfp963	-0.71	1.19E-04	E130119H09Rik	-0.84	2.09E-03
Emr1	-0.66	6.87E-06	Ttc21a	-0.63	1.20E-04	Tecrl	-1.14	2.12E-03
Slc22a26	-0.87	7.01E-06	Tmem88	-0.68	1.22E-04	D930020B18Rik	-0.67	2.12E-03
Gm9873	-0.68	7.30E-06	Gm17455	0.62	1.24E-04	LOC102635387	-0.72	2.13E-03
D4Ertd199e	-0.84	7.35E-06	Cenpm	-0.65	1.26E-04	Gm11691	-0.62	2.15E-03
C030004M13Rik	-0.75	7.39E-06	Prdm16	-0.60	1.28E-04	Cd209d	-0.67	2.15E-03

Fgf10	-0.65	7.48E-06	Hoxa11	-0.97	1.31E-04	Tpd52l1	0.77	2.18E-03
Gyltl1b	-0.86	7.58E-06	4921524M04Rik	0.65	1.31E-04	AW124847	-0.59	2.21E-03
4933416O17Rik	-0.59	8.15E-06	Pcdhb19	-0.60	1.35E-04	Slc18a3	-0.66	2.25E-03
4930442P07Rik	0.79	8.23E-06	Pf4	-0.93	1.36E-04	Hpdl	-0.60	2.25E-03
LOC102632821	-0.73	8.38E-06	Stra6	-1.43	1.36E-04	1700001G11Rik	-0.72	2.27E-03
AI451250	-0.84	8.71E-06	Foxd3	-0.62	1.36E-04	4931408D14Rik	-0.65	2.27E-03
Cbx3	-0.71	8.75E-06	Crim1	-0.78	1.38E-04	Tmsb15a	-0.67	2.29E-03
8030423J24Rik	-0.72	8.84E-06	Mesp1	-0.71	1.41E-04	Nfkbil1	-0.82	2.32E-03
4930442J19Rik	-0.79	8.89E-06	Alox12e	0.77	1.50E-04	B230311B06Rik	-0.61	2.37E-03
Slamf9	-0.76	9.17E-06	Tprn	-0.78	1.50E-04	C86090	0.68	2.37E-03
Prrxl1	-0.60	9.19E-06	Vmn2r26	-0.64	1.51E-04	D10Ertd761e	-0.67	2.44E-03
Bmp6	-0.89	9.24E-06	BC030867	-0.63	1.54E-04	Neurog2	-0.66	2.49E-03
Gm7544	-0.74	9.27E-06	1700020L24Rik	-0.62	1.55E-04	Cenpi	-0.75	2.49E-03
D18Ertd169e	-0.94	9.42E-06	Trim15	-0.83	1.59E-04	B430306N03Rik	-0.64	2.58E-03
Tsga8	-0.64	9.70E-06	Aebp1	-0.70	1.59E-04	Zfp37	-0.63	2.62E-03
Lgals7	-0.59	9.89E-06	LOC102640815	-0.65	1.60E-04	Gm13262	-0.63	2.64E-03
5830485P09Rik	0.66	9.96E-06	AI467606	-0.60	1.64E-04	Ccdc81	-0.62	2.66E-03
Efcab1	-0.66	1.00E-05	C77267	-0.86	1.65E-04	4930552P12Rik	-0.59	2.68E-03
Slc4a1	-0.74	1.01E-05	Ip6k3	-0.76	1.66E-04	AU015148	-0.66	2.69E-03
Zcwpw2	0.60	1.06E-05	Tex35	-0.65	1.70E-04	Pld6	-0.69	2.72E-03
C130090I23Rik	-0.69	1.08E-05	Sult2a2	-0.64	1.70E-04	Ercc6	-0.64	2.86E-03
Dll4	-0.75	1.09E-05	Synm	-0.64	1.71E-04	Dynlt1f	-0.75	2.96E-03
4930578M01Rik	-0.63	1.10E-05	A430105J06Rik	-0.79	1.73E-04	Fbxl7	-1.32	3.12E-03
Rab32	-0.63	1.11E-05	Osbpl7	-0.72	1.74E-04	Ctcflos	-0.61	3.14E-03
Rilp	0.68	1.11E-05	LOC102635622	1.06	1.77E-04	Atp6v1e2	-0.75	3.28E-03
Tgm1	0.63	1.12E-05	Mtnr1a	-0.70	1.79E-04	Mrgpra3	-0.68	3.29E-03
Tor4a	0.72	1.13E-05	8430418B16Rik	-0.59	1.83E-04	Cd97	-0.59	3.31E-03
6330565B04Rik	-0.61	1.14E-05	Jade3	-0.88	1.83E-04	Pon1	-1.89	3.45E-03
Zfp873	-0.62	1.16E-05	Tm4sf4	-0.65	1.87E-04	Becn2	-0.64	3.50E-03
B930053N05Rik	0.72	1.18E-05	C77651	-1.08	1.87E-04	LOC102636612	-0.72	3.56E-03
BC026600	-0.83	1.21E-05	Zfp324	-1.37	1.94E-04	A130033P14	-0.60	3.59E-03
Ly86	-0.89	1.23E-05	5730522E02Rik	-0.84	2.06E-04	Zfp605	-1.00	3.59E-03
St6galnac4	-0.85	1.27E-05	Sult1c2	-0.73	2.07E-04	Tex37	-0.72	3.61E-03
Gjb5	-0.59	1.27E-05	Akr1e1	-0.84	2.11E-04	Cpxm2	-0.97	3.68E-03
Mycbpap	-0.62	1.28E-05	Igfbp6	-1.06	2.11E-04	Brpf3	-0.74	3.70E-03
C330022C24Rik	-0.96	1.29E-05	Chst9	0.60	2.13E-04	Mroh2a	-0.59	3.73E-03
4933423P22Rik	-0.71	1.29E-05	Prss21	-0.59	2.16E-04	9430065F17Rik	-0.65	3.82E-03
Plk1	-0.80	1.30E-05	Iqgap2	-0.76	2.23E-04	Ptgis	-0.64	3.93E-03
Arhgap20os	0.86	1.42E-05	Alpi	-0.67	2.28E-04	BC001981	0.61	3.94E-03
Cass4	-0.69	1.43E-05	C530045E16Rik	-0.88	2.37E-04	Gramd1c	0.59	3.97E-03
AU021760	-0.59	1.46E-05	Slc16a9	-0.63	2.44E-04	G530011O06Rik	1.26	3.97E-03

4930556L07Rik	-0.59	1.50E-05	4930442P19Rik	-1.03	2.44E-04	Acox2	-0.61	4.02E-03
Kank4	-1.25	1.50E-05	2900092C05Rik	-0.72	2.46E-04	Fap	-1.08	4.05E-03
Thbs4	-0.77	1.51E-05	4930438A08Rik	-0.67	2.47E-04	Gm12758	0.61	4.13E-03
M1ap	-0.62	1.53E-05	C030034I22Rik	-1.11	2.47E-04	Mia	0.86	4.17E-03
4933433G15Rik	0.70	1.57E-05	Ptgdr	0.65	2.50E-04	C77534	0.97	4.22E-03
Tas1r3	-0.94	1.58E-05	Fv1	-1.23	2.55E-04	Tmem229b	-0.63	4.27E-03
Arhgef3	-0.93	1.59E-05	Khdc3	-1.27	2.56E-04	Zfp229	-0.60	4.32E-03
Klrg2	-0.81	1.63E-05	S100a4	0.62	2.57E-04	Gm10796	0.72	4.36E-03
Themis2	-0.62	1.70E-05	Gm9798	-0.76	2.67E-04	4930525G20Rik	-0.64	4.43E-03
4933413J09Rik	-0.68	1.71E-05	Adad2	-0.69	2.67E-04	Rnf222	-0.59	4.64E-03
Neu2	-0.74	1.74E-05	Icam2	-1.12	2.73E-04	Pmaip1	0.70	4.68E-03
Prl2a1	-0.71	1.75E-05	Vwa7	0.64	2.75E-04	4930417H01Rik	-0.77	4.69E-03
Cdrt4	-0.76	1.76E-05	1700012B07Rik	-0.64	2.75E-04	Plac8	-0.59	4.71E-03
Gpr27	-0.64	1.77E-05	Zfp113	-0.80	2.76E-04	5830443J22Rik	-0.60	4.78E-03
Vnn3	-1.05	1.77E-05	Clrn1	0.66	2.81E-04	Cep250	-1.14	4.97E-03
Orm3	-0.78	1.79E-05	2810040C05Rik	-0.64	2.86E-04	Slc16a8	-0.73	5.17E-03
BC005561	-0.73	1.79E-05	Mef2b	-0.67	2.86E-04	E030042O20Rik	-0.64	5.24E-03
D9Ertd26e	-1.01	1.88E-05	D17892	-0.60	2.86E-04	Maml2	-0.67	5.49E-03
Zfp524	-0.69	1.94E-05	Lims2	-0.76	2.89E-04	Hdhd1a	-0.66	5.65E-03
Fbp2	-0.74	1.94E-05	Nlrp6	-1.32	2.90E-04	Itpripl2	0.64	5.76E-03
Pik3cb	0.71	1.94E-05	Itih1	-0.64	2.90E-04	Mogat1	-0.86	5.81E-03
Fancb	-0.64	1.95E-05	Aqp5	0.77	2.95E-04	4930417O22Rik	-0.61	5.83E-03
Hrh2	-0.85	2.01E-05	Tnmd	-1.60	3.03E-04	Aldh3a1	0.62	6.01E-03
Sowaha	-0.63	2.01E-05	4930483K19Rik	-0.69	3.06E-04	Slc6a13	-0.77	6.13E-03
4933407G14Rik	-0.71	2.02E-05	Ccdc13	-0.84	3.11E-04	Ehhadh	-0.68	6.17E-03
Fam216b	-1.02	2.04E-05	2010001K21Rik	-0.60	3.36E-04	C80283	-0.67	6.27E-03
1700023B13Rik	-0.62	2.06E-05	Myrf	-0.86	3.38E-04	Cetn4	0.65	6.28E-03
Map3k6	-0.71	2.07E-05	Gdf15	-0.72	3.48E-04	Cryaa	-0.86	6.30E-03
4933434C23Rik	-0.81	2.12E-05	Sprr2k	-1.03	3.62E-04	E330022O07	-0.75	6.33E-03
A430010J10Rik	-0.64	2.12E-05	Myoc	-0.64	3.63E-04	AU019157	-0.65	6.69E-03
C430042M11Rik	-2.32	2.15E-05	1700001L05Rik	-0.98	3.67E-04	Krt8	-0.85	6.92E-03
1700034E13Rik	-0.68	2.21E-05	Cd209a	-0.93	3.70E-04	Ifi27l2a	-1.88	6.94E-03
Rarres1	-0.77	2.26E-05	Sh3tc2	-0.61	3.72E-04	Bub1b	-0.70	7.11E-03
Hoxb3	-0.63	2.31E-05	4930403D09Rik	-0.59	3.80E-04	BC048562	-0.62	7.19E-03
LOC102638854	-0.65	2.35E-05	Dus4l	-0.69	3.82E-04	6330407A03Rik	0.83	7.24E-03
AF067063	0.60	2.37E-05	9430081I23Rik	-0.59	3.85E-04	Sema3c	-0.71	7.25E-03
Sh3d21	-0.72	2.43E-05	4930550C17Rik	-0.84	3.94E-04	Dhrs7c	-0.64	7.37E-03
Gadd45gip1	-0.69	2.47E-05	Uts2r	-0.62	3.95E-04	Car12	-0.85	7.42E-03
Rbfa	-0.77	2.52E-05	Maff	-0.65	3.95E-04	H2-Aa	-0.89	7.46E-03
1700023G09Rik	0.68	2.55E-05	Ttc36	-0.59	4.06E-04	Gpr133	-0.61	7.68E-03
Cldn26	-0.77	2.61E-05	Mrgprg	-0.64	4.11E-04	Ccne2	-0.60	7.77E-03

Gm12185	-0.70	2.62E-05	Pdzd3	-0.59	4.16E-04	Gpt2	0.59	7.95E-03
D830025C05Rik	-0.70	2.63E-05	LOC102636933	-0.86	4.19E-04	Entpd8	-0.60	8.16E-03
Ropn1	-0.85	2.65E-05	2310061G22Rik	-0.60	4.20E-04	Cebpa	-0.67	8.34E-03
4930455D15Rik	-0.71	2.68E-05	Casq2	-0.64	4.38E-04	Ttr	-1.26	8.45E-03
Gja10	-0.63	2.79E-05	4930481A15Rik	-0.62	4.45E-04	Slc6a20a	-1.80	8.45E-03
Phlda2	-0.71	2.85E-05	9230110K08Rik	-0.81	4.50E-04	Gm9799	-0.62	8.85E-03
Myl4	-0.72	2.85E-05	1700018B24Rik	-1.01	4.56E-04	Col1a1	-0.81	9.66E-03
Thsd4	-0.63	2.85E-05	4933402J07Rik	0.78	4.77E-04	Rgr	-1.65	9.67E-03
Glod5	-0.60	3.00E-05	Wnt10a	-0.90	4.79E-04	C4bp-ps1	-0.61	9.98E-03
Lyrm7	0.69	3.05E-05	Myl7	-0.60	4.79E-04	D14Ertd426e	-0.62	1.01E-02
Fshr	-0.81	3.06E-05	Ccdc162	-0.80	4.87E-04	Olfml3	-0.98	1.11E-02
Pmch	-0.95	3.19E-05	Cib3	-0.90	4.91E-04	Cyp2d40	-0.68	1.18E-02
AF067061	-0.79	3.26E-05	4930555F03Rik	0.62	4.94E-04	Ermap	-1.24	1.22E-02
Six2	-0.59	3.26E-05	Zfp3	-0.64	4.97E-04	5730507C01Rik	-0.61	1.23E-02
Mmp11	0.63	3.28E-05	Slc39a12	-0.64	4.98E-04	Rbm45	0.73	1.24E-02
Tac4	-0.65	3.28E-05	Hmgcs2	-0.80	5.11E-04	D3Ertd740e	-0.70	1.25E-02
Krt31	-0.98	3.35E-05	Cdh3	-0.59	5.19E-04	D430013B06Rik	-0.72	1.26E-02
Ifi47	-0.73	3.37E-05	4933416A02Rik	-0.76	5.46E-04	Oca2	-0.67	1.26E-02
4933411K16Rik	-0.64	3.43E-05	Fbxo17	-0.75	5.46E-04	Hp	-0.70	1.27E-02
Il21r	-0.59	3.43E-05	5730409L17Rik	-0.79	5.53E-04	Gm6712	0.71	1.29E-02
Murc	-0.88	3.46E-05	Phf5a	-0.61	5.58E-04	Spert	-0.61	1.30E-02
Slitrk4	0.65	3.52E-05	Frmd3	-0.85	5.59E-04	Padi4	-2.61	1.37E-02
Phc3	-0.62	3.61E-05	Syce3	-0.63	5.64E-04	Col24a1	1.14	1.39E-02
Myl6b	-0.64	3.73E-05	Akr1c13	-0.60	5.86E-04	Edn2	0.59	1.42E-02
Cldn15	-0.73	3.85E-05	Gm10575	-0.63	5.87E-04	Smc2os	-0.66	1.46E-02
Pbx4	-0.63	3.91E-05	1700109H08Rik	0.62	5.99E-04	4930505N22Rik	0.62	1.46E-02
C87926	-0.62	3.92E-05	Tssk5	-0.60	6.02E-04	Npl	1.23	1.53E-02
C76434	0.82	3.97E-05	Prss30	-0.59	6.26E-04	Ifi27l2b	-0.68	1.59E-02
Lyve1	-0.88	4.02E-05	Tescl	-0.88	6.33E-04	Zfp133-ps	1.28	1.65E-02
Wisp2	-0.70	4.07E-05	2510019K15Rik	-0.65	6.50E-04	Rpe65	-1.70	1.72E-02
Olfr1508	0.64	4.17E-05	Mmp8	-0.60	6.60E-04			
